# Challenging the Hypothesis of in Utero Microbiota Acquisition in Healthy Canine and Feline Pregnancies at Term: Preliminary Data

**DOI:** 10.3390/vetsci10050331

**Published:** 2023-05-04

**Authors:** Penelope Banchi, Barbara Colitti, Andrea Del Carro, Michela Corrò, Alessia Bertero, Ugo Ala, Angela Del Carro, Ann Van Soom, Luigi Bertolotti, Ada Rota

**Affiliations:** 1 Department of Veterinary Sciences, University of Turin, 10095 Grugliasco, Italy; barbara.colitti@unito.it (B.C.); alessia.bertero@unito.it (A.B.); ugo.ala@unito.it (U.A.); angela.delcarro@unito.it (A.D.C.); luigi.bertolotti@unito.it (L.B.); ada.rota@unito.it (A.R.); 2 Department of Internal Medicine, Reproduction and Population Medicine, Faculty of Veterinary Medicine, Ghent University, 9820 Merelbeke, Belgium; ann.vansoom@ugent.be; 3 Iunovet-Clinique Vetérinaire Saint Hubert, 06240 Beausoleil, France; andrea.delcarro@gmail.com; 4 Istituto Zooprofilattico Sperimentale delle Venezie, 35020 Legnaro, Italy; mcorro@izsvenezie.it

**Keywords:** feto-maternal microbiota, dog, cat, fetus, pregnancy

## Abstract

**Simple Summary:**

This preliminary study aimed to assess the presence of bacteria in pregnancy tissues belonging to healthy canine and feline feto-maternal units at term. Specifically, it included bitches and queens undergoing elective cesarean sections and their first extracted fetus. The placental side of the endometrium, amniotic fluid, and meconium were sampled as tissue representative of the intra-uterine environment during pregnancy. Sampling and laboratory protocols were elaborated to contrast the possibility for contamination and included strict selection criteria (only elective cesarean sections, no recent treatments with antimicrobials), sterility during sample collection, and sampling and laboratory controls. Samples were processed using both culture and molecular techniques (16S rRNA bacterial gene sequencing). When positive, culture revealed the presence of bacteria that are common contaminants and sequencing yielded a very low bacterial load. A difference was highlighted between canine and feline samples, suggesting a possible contamination from the skin of the dam, although the small sample size prevents any definitive conclusion. This study suggests that healthy canine and feline fetuses might develop in the presence of low amounts of bacterial components, although future research should include stricter protocols to check for contamination and provide information on bacterial viability.

**Abstract:**

At present, there are no data on the presence of bacteria in healthy canine and feline pregnancies at term. Here, we investigated the uterine microbiome in bitches (*n* = 5) and queens (*n* = 3) undergoing elective cesarean section in two facilities. Samples included swabs from the endometrium, amniotic fluid, and meconium, and environmental swabs of the surgical tray as controls. Culture and 16S rRNA gene sequencing were used to investigate the presence of bacteria. Culture was positive for 34.3% of samples (uterus *n* = 3, amniotic fluid *n* = 2, meconium *n* = 4, controls *n* = 0), mostly with low growth of common contaminant bacteria. With sequencing techniques, the bacterial abundance was significantly lower than in environmental controls (*p* < 0.05). Sequencing results showed a species-specific pattern, and significant differences between canine and feline bacterial populations were found at order, family, and genus level. No differences were found in alpha and beta diversities between feto-maternal tissues and controls (*p* > 0.05). Dominant phyla were Bacteroidetes, Firmicutes, and Proteobacteria in different proportions based on tissue and species. Culture and sequencing results suggest that the bacterial biomass is very low in healthy canine and feline pregnancies at term, that bacteria likely originate from contamination from the dam’s skin, and that the presence of viable bacteria could not be confirmed most of the time.

## 1. Introduction

It is a long-standing dogma that a sterile uterine environment is necessary to carry a healthy pregnancy to term. This theory is known as the ‘sterile womb paradigm’ [1] and it has been questioned in recent years, since bacterial communities were identified in samples from feto-maternal units (placenta, amniotic fluid, and/or meconium) of humans [2,3,4,5,6] and animals (mice, ruminants, dogs, and horses) [6,7,8,9]. This supports the hypothesis that the neonatal microbial colonization starts prior to birth. However, other studies failed to detect consistent evidence of bacterial presence both in healthy human [10,11,12,13] and animal [14,15,16] pregnancies, using molecular-based culture-independent and culture-dependent techniques alone or in combination. A recent perspective [17] led to reject the in utero colonization hypothesis in humans, showing the pitfalls of DNA sequencing on ‘low-’ and ‘zero-biomass’ samples, as feto-maternal samples are often classified. Nevertheless, the species specificity of the feto-maternal connection and the high heterogenicity of studies assessing the feto-maternal microbiome in animals do not allow us to draw definitive conclusions on non-human models, and this topic remains controversial. Adequate sampling procedures and processing are fundamental when dealing with low-biomass samples with the primary aim of avoiding contamination [18].

To the authors’ knowledge, no data are available on feline pregnancies and only two studies were carried out in dogs [8,19], both including puppies born either through natural birth or emergency cesarean (C) section. During natural birth, the fetus is colonized by the dam’s vaginal flora, whereas in case of emergency C-section, the cervix is normally open, allowing for in-uterus migration of bacteria. Hence, only planned elective C-sections in the presence of a closed cervix can guarantee the microbiological isolation of the pregnant uterine environment at term. Furthermore, previous results in dogs were only based on culture-dependent investigation [8,19]. Although traditional culture allows for the assessment of the viability of the isolated bacteria, it fails to detect those microorganisms that cannot grow in culture [20] and false negatives are not uncommon when the bacterial load is extremely low, as can be expected for feto-maternal samples [17,21].

The objective of the present research was to test the in utero colonization hypothesis in healthy canine and feline pregnancies at term, during elective C-sections, using both culture-dependent and culture-independent techniques.

## 2. Materials and Methods

### 2.1. Animals

Five bitches and three queens that underwent elective C-section at the Veterinary Teaching Hospital of the University of Turin (Italy) or at a private practice (Iunovet-Clinique Vetérinaire Saint Hubert, Beau-soleil, France) were enrolled in this research. Each dam and her fetuses were considered as one feto-maternal unit. No restrictions were placed on the breed, sex, age, parity, or weight of the animals. A C-section was planned due to breed predisposition to dystocia (e.g., brachycephalic breeds) [22] or previous C-sections [23].

Healthy bitches and queens having uneventful pregnancies were included after a full clinical examination and ultrasound evaluation to assess fetal viability. Specifically, the bi-parietal diameters were measured, and the presence of intestinal peristalsis was verified to assess gestational age and to verify the maturity of the fetuses, respectively [24]. Blood progesterone concentration was measured in bitches to confirm cervical closure before the onset of the first stage of parturition, since a serum progesterone concentration lower than 2 ng/mL was found to be associated with an open cervix [25]. Drug administration (i.e., antimicrobial agents and corticosteroids) in the past six months and the presence of pregnancy complications (e.g., dead fetuses, dystocia), were considered as exclusion criteria.

This research was performed in agreement with the ARRIVE guidelines (Animal Research: Reporting In Vivo Experiments) [26,27], which were specifically adjusted for this type of observational study. This research has been approved by the Ethical Committee of the Department of Veterinary Sciences of the University of Turin (Italy) (N. 66/10/01/2020 and n. 310/9/2/2021). All the owners provided informed written consent and the procedures were carried out in accordance with the EU Directive 86/609/CEE and with the guidelines of the Italian Ministry of Health for the care and use of animals (D.L. 4 March 2014 n. 26 and D.L. 27 January 1992 n. 116).

### 2.2. Cesarean Sections and Sample Collection

Animals were prepared for surgery according to standard procedures, in the pre-operative room. General anesthesia was induced inside the surgical room with propofol i.v. (Proposure, Boehringer Ingelheim Animal Health, Milano, Italy), and maintained with isoflurane (Iso-Vet 1000 mg/mL, Piramidal Critical Care Italia, San Giovanni Lupatoto, Italy) after intubation. The surgical area was scrubbed with three passages of two different antiseptic solutions (70% ethanol and 2% povidone iodine) and sterile surgical drapes were placed around the surgical site.

C-sections were always performed following standard procedures [8] and samples were collected concurrently by an operator other than the surgeon. Only the first extracted fetus was sampled to (1) limit possible environmental contamination related to the time of exposure of the uterine content, (2) avoid prolonging surgical time, and (3) immediately extract all the other fetuses and resuscitate them.

Briefly, after opening the abdomen, the uterus was slowly exposed, and a single incision was performed at the base of a uterine horn. Two sterile nylon regular swabs (ESwab 480CE, Copan Italia Spa, Brescia, Italy) were used to sample the site where the placenta of the first extracted fetus was attached to the endometrium. One swab was placed into a 5 mL tube containing 1 mL of modified Liquid Amies Medium (ESwab^®^ Copan Italia Spa, Brescia, Italy) for bacterial culture. The other swab was cut with sterile scissors and stored in a sterile Eppendorf tube (Eppendorf Tubes^®^ 3810X, Eppendorf s.r.l., Hamburg, Germany) for molecular analyses. Samples of amniotic fluid were also collected from the closed amniotic sac of the first extracted fetus using a sterile syringe and a sterile 20 G needle. The amniotic fluid was dropped onto a sterile nylon regular swab (ESwab 480CE, Copan Italia Spa, Brescia, Italy) for culture and 1 mL was poured in an Eppendorf tube for molecular analyses. The fetus was passed to an assistant wearing sterile gloves and placed on a surgical tray covered with sterile surgical drapes, fetal membranes were opened, and resuscitation of the fetus was started by rubbing it with sterile drapes. Once the puppy was resuscitated, two samples of meconium from the rectal ampulla were collected using two ‘mini’ swabs (ESwab 484CE, Copan Italia Spa, Brescia, Italy), one for culture and one for molecular analyses. Meanwhile, the C-section was completed. Cefazolin (20 mg/kg i.v., Teva, Italy) was administered to the dam after the extraction of the first fetus. Two regular swabs of the same type used for sampling (one for culture and one for molecular analyses) were left open on the surgical tray throughout the surgical procedure as environmental controls.

All the samples collected for bacterial culture were immediately sent to the Istituto Zooprofilattico Sperimentale delle Venezie (Legnaro, Italy) and processed within 48 h, whereas all the samples for metagenomic analysis were frozen at −80 °C and processed at the same time.

### 2.3. Bacterial Culture

Bacterial isolation was performed as previously reported [8], following standard laboratory procedures [12]. Briefly, after dilution of the swabs in 1 mL of nutritive broth (Heart Infusion Broth, HIB, Conda, Madrid, Spain), different solid and liquid media were inoculated with 10 µL and 100 µL of the suspension and incubated in different atmospheric conditions. The presence of aerobic microorganisms was assessed using nutrient blood agar (Blood Agar Base n° 2, BA, Biolife, Milan, Italy) with 5% sterile defibrinated sheep blood (Allevamento Blood, Teramo, Italy), McConkey agar (Thermo Fisher Oxoid Ltd., Basingstoke, UK) as a selective medium for *Enterobacteriaceae*, and bile-esculin azide agar, BEA (Conda, Madrid, Spain). After inoculation, the media were incubated under aerobic conditions at 37 °C ± 1 °C for 24 h. The presence of anaerobic microorganisms was performed via inoculation in nutrient blood agar, BA medium, selective medium for *Clostridium perfringens*, PBA (TSC Agar Base with specific *C. perfringens* selective supplement, Biolife, Milan, Italy) added with 5% sterile defibrinated sheep blood, and fluid thioglycolate medium (Liofilchem, Teramo, Italy). Incubation lasted 48 h at 37 °C ± 1 °C, and anaerobic conditions were obtained by transferring the inoculated media into jars equipped with an anaerobic generating system and the anaerobic indicator AnaeroGen 2.5 L/3.5 L/Compact—Atmosphere Generation System and Anaerobic indicator BR0055b (Thermo Fisher Oxoid Ltd., Basingstoke, UK).

The growth of aerobic and anaerobic bacteria was assessed after 24 and 48 h, respectively. In case of lack of growth of bacteria on agar media in combination with turbidity of the nutrient broth, the plates were further incubated for an additional 24 or 48 h.

Bacterial growth was estimated by counting the number of colony-forming units (CFUs) in the first isolation plates and classified according to the standard laboratory operating procedures of the laboratory performing the analysis. Specifically, bacterial growth was considered as low (1–10 CFU/10 µL), moderate (11–30 CFU/10 µL),), or high (≥31 CFU/10 µL).

Macroscopic observation of colonies, Gram stain, cellular morphology, growth on selective medium, catalase, oxidase, mobility tests, and coagulase tube test were used for the identification of the bacterial genera. Bacterial species identification was carried out via MALDI-TOF MS: Microflex LT instrument (MALDI Biotyper, Bruker Daltonics, Billerica, MA, USA) using the FlexControl software (version 3.3, Bruker Daltonics, Billerica, MA, USA). “Highly probable species identification” was considered when scores were higher than 2.3; scores between 2 and  2.299 indicated “secure genus identification and probable species identification”, scores between 1.7 and  1.999 indicated “probable genus identification”, and scores lower than 1.7 indicated “unreliable identification” [28].

### 2.4. Sequencing of 16S rRNA Gene

Bacterial genomic DNA from the animal samples (*n* = 24) and corresponding negative environmental and laboratory controls (*n* = 16) was extracted using RNeasy Power Microbiome KIT (Qiagen, Hilden, Germany) according to the manufacturer’s instructions. One microliter of RNaseA (Thermo Fisher Scientific, Waltham, MA, USA) was added to digest RNA, with an incubation of 1 h at 37 °C. DNA was quantified with a fluorimetric method Qubit High Sensitive dsDNA kit (Life Technologies, Carlsbad, CA, USA) and standardized at 5 ng/μL.

The 16S rRNA gene was amplified following the Illumina 16S Metagenomic Sequencing Library Preparation Protocol (Illumina Inc. San Diego, CA, USA), with some modifications. Briefly, the V3-V4 region of the 16S gene was amplified with unique barcoded PCR primers containing the Illumina adapter overhang nucleotide sequences: 16S Forward Primer (5′- TCGTCGGCAGCGTCAGATGTGTATAAGAGACAGCCTACGGGNGGCWGCAG) and16S Reverse Primer (5′-GTCTCGTGGGCTCGGAGATGTGTATAAGAGACAGGACTACHVGGGTATCTAATCC). PCR amplicons were cleaned up using NucleoMag^®^ NGS Clean-up and Size Select (Macherey-Nagel, Allentown, PA, USA) following the double size selection protocol. The resulting products were tagged by using the Nextera XT Index Kit (Illumina Inc., San Diego, CA, USA). After the second purification step, amplicon products were quantified using Qubit High Sensitive dsDNA kit (Life Technologies, Carlsbad, CA, USA). Purified and normalized libraries were then pooled and diluted to a 4 nM concentration. The pooled library was then denatured with 0.2 N NaOH, diluted to 10 pM, and combined with 20% (vol/vol) denatured 10 pM PhiX, prepared according to Illumina guidelines. The sequencing was performed on the MiSeq Illumina platform (Illumina Inc., San Diego, CA, USA) with V3-600 cycles chemistry.

### 2.5. Data Analysis

The analysis, including classification of the sequences in OTUs (Operational Taxonomic Unit) with 97% similarity and taxonomy assignment, was performed in Qiime2 [29], following the Qiime2 standard operating procedure for MiSeq data.

Specifically, paired-end reads were assembled and assigned to their original sample based on the barcode. Primer sequences and barcodes were then removed. For the denoising procedure, the Deblur method implemented in Qiime2 was used. Alpha diversity and Beta diversity were measured using Qiime2, as well as all the core metrics. Taxonomy was retrieved using BLASTn rRNA typestrains; a 16S ribosomal RNA database and sample bacterial population was assessed using hand-made R procedures. Data analyses were further conducted using R ver. 4.2.2 (Vienna, Austria). Differences among samples were calculated using alpha (Shannon and Faith indexes) and beta diversity estimation, based on Jaccard, Bray–Curtis and Unweighted Unifrac distance matrices, with a Qiime pipeline. Finally, we considered the presence/absence of bacterial strains. In more detail, we evaluated Pearson’s correlation index between paired samples using the quantitative presence/absence patterns of bacterial strains at all the taxonomical levels (order, family, genus, species; dist.binary function in R). Cluster analysis was conducted drawing complete-linkage dendrograms and reconstructing population structure space using two-dimensions scaling plots (cmdscale function in R). Population subdivisions and clusters were evaluated using the k-means clustering approach [30]. The association between the population structure and the samples features was evaluated using the Chi-squared test and Fisher’s exact test. In particular, the association (k means function in R) was evaluated between the evaluated clusters membership and animal species (K = 2 for k-means clustering evaluation), surgical facility (K = 2), feto-maternal units (K = 5 for dogs, K = 3 for cats), feto-maternal tissues (K = 4, for dogs and cats separately). Moreover, we evaluated the differences between feto-maternal tissues and controls (K = 2).

## 3. Results

The mean and standard deviation (SD) for age, weight, and litter size along with the list of breeds of the bitches and queens included in the study are reported in Table 1. One bitch was primiparous, whereas four bitches were pluriparous. All the queens were primiparous.

A total of 25 puppies and 10 kittens were born through cesarean section. Twenty-four puppies were alive and one stillborn puppy was extracted from the uterus of the bitch with the largest litter size (*n* = 7). All kittens were alive. This study therefore includes five canine feto-maternal units (FMU) and three feline FMU.

### 3.1. Canine Feto-Maternal Units: Bacterial Culture

The results of the bacterial culture are reported in Table 2. Two canine feto-maternal units (C-FMU-1 and C-FMU-4) were negative. Three out of five meconium samples resulted in bacterial growth. Additionally, two uterine samples and one amniotic fluid sample showed positive culture results. C-FMU-2 was the only one to show bacterial growth for all samples, although bacterial species were different in-between samples (*Bacillus* spp. in the uterus and amniotic fluid; *Staphylococcus hominis* and *Acinetobacter baumannii* in the meconium).

Pure cultures were obtained for all the positive samples, except for the meconium of the C-FMU-2, presenting substantial growth of *Staphylococcus hominis* and *Acinetobacter baumannii*. The same FMU (C-FMU-2) was the only one in which positivity was recorded for all the samples and growth was reported as high; differently from meconium, the amniotic fluid and the uterine sample were positive for *Bacillus* spp. *Pseudomonas fluorescens* was isolated from the uterine sample of C-FMU-5 (low growth), whereas *Acinetobacter baumannii* presented high growth when the meconium belonging to the same C-FMU was investigated. Microorganisms belonging to the genus *Clostridium* or other strict anaerobic bacteria were never isolated. Controls were always negative.

### 3.2. Feline Feto-Maternal Units: Bacterial Culture

The results of the bacterial culture are reported in Table 3. The culture of one feline feto-maternal unit (F-FMU-1) was negative. In the two feto-maternal units presenting positive results, only pure cultures were obtained. Specifically, two uterine samples, one amniotic fluid sample, and two meconium samples showed bacteria growth. High growth (≥31 CFU/10 µL) was recorded only for one uterine sample (F-FMU-3), whereas all the other positive samples showed very low bacterial growth.

*Pseudomonas aeruginosa* was the bacterium isolated from the uterine sample that showed high growth (F-FMU-3). The other positive uterine culture showed a few colonies of a coagulase-negative Staphylococcus (C-FMU-2). *Pseudomonas aeruginosa* was the only bacterium isolated from the amniotic fluid of F-FMU-2, although in very low numbers. Finally, few colonies of *Psychrobacter sanguinis* were isolated from the meconium of F-FMU-3. Microorganisms belonging to the genus *Clostridium* or other strict anaerobic bacteria were never isolated. Controls were always negative.

### 3.3. Canine and Feline Feto-Maternal Units: Sequencing of 16s RNA

A total of 31 samples were sequenced using Illumina MiSeq platform. Specifically, all animal samples (except for one feline amniotic fluid sample from F-FMU-1) and all environmental samples were sequenced. Laboratory controls and the amniotic fluid from F-FMU-1 did not produce sufficient amplification for further processing. The Qiime protocol identified a total of 239 different features (i.e., unique 16S rRNA sequences). Among the 31 available samples, the median number of features was 912 (range: 2–91,866). The features abundance was significantly higher in controls compared to animal ones (controls median = 16,866.5, IQR = 20,951; feto-maternal samples median = 587, IQR = 909; Wilcoxon test *p* < 0.05). The rarefaction curve evaluated on both Shannon and Faith indexes showed a plateau for all grouping methods (samples, species, group, and tissue).

The dominant phyla in the amniotic fluid of both species were *Bacteroidetes* and *Firmicutes*. A match was present in dominant phyla between dogs and cats, with *Firmicutes*, *Proteobacteria*, and *Bacteroidetes* being the most prevalent. *Proteobacteria* and *Bacteroidetes* were the most represented phyla in canine meconium. The latter was the most dominant in feline meconium, followed by *Firmicutes*. As for the controls, *Firmicutes*, *Proteobacteria*, and *Bacteroidetes* were the prevalent phyla during the sampling of both species. Within- and among-group diversities (alpha and beta diversity) were evaluated using the Qiime standard pipeline. No differences were found in Shannon indexes when animal species or tissues were compared (Wilcox test and Kruskal–Wallis test *p* > 0.05, respectively). When among-groups differences were evaluated, beta diversity did not reveal any statistically supported differences among samples for all the used methods (Jaccard, Bray–Curtis and unweighted UniFrac distance matrices; Permanova test *p* > 0.05).

**Figure 1:**
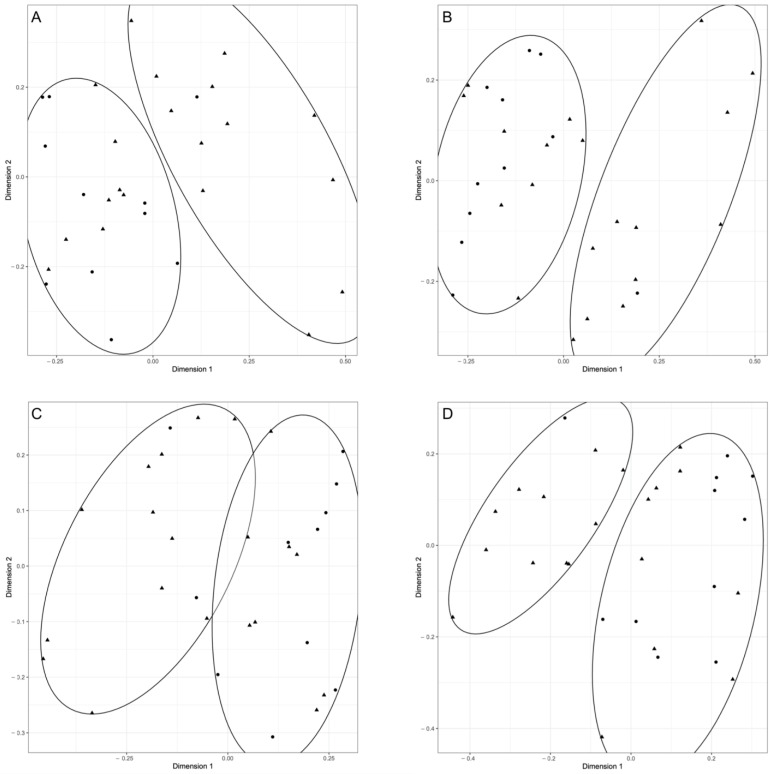
Two-dimension plot based on Pearson’s correlation index evaluated based on the bacterial presence/absence patterns of each sample. Ellipses represent the clusters evaluated using k-means function with K = 2 for animal species comparison. Circles represent canine samples; triangles represent feline samples. The four panels show the clustering of samples at all the considered taxonomical levels ((**A**): order; (**B**): family; (**C**): genus; (**D**): species).

Patterns of the presence/absence of each bacterial strain were calculated for the 31 sequenced samples and Pearson’s correlation index was calculated to construct a distance matrix and to depict two-dimensional scaling plot for the four taxonomic levels (order, family, genus, species). The K means approach was used to independently identify the clusters within the bacterial population structure obtained with the two-dimensional scaling reconstruction at each taxonomical level. The association between cluster membership and grouping methods was reported in Table 4. The only statistically significant association is obtained when animal species are considered for the grouping method (at order, family, and species levels). As shown in Figure 1, clusters always discriminate between feline and canine samples. Interestingly, association analysis revealed no differences between animal samples and controls. Finally, a slight difference among dog tissues was recorded, only at the bacterial family taxonomical level.

## 4. Discussion

This preliminary study investigated the feto-maternal microbiota of two domestic animal species (i.e., dogs and cats) using culture-dependent and culture-independent methods, with the objective of testing the in utero colonization hypothesis and setting the stage for future research.

Strict asepsis measures were adopted to minimize the chances of contamination, and only elective C-sections were included before the onset of the first stage of parturition, when the cervix was still closed, sealing the uterus from the vaginal lumen. Elective C-sections are rather infrequent in cats, and this led to the inclusion of only three feline feto-maternal units; however, this study represents pioneering research in this field in cats. In a previous study in dogs [8], the presence of bacteria was investigated solely using culture; microorganisms were detected in the placenta, amniotic fluid, and meconium, although both elective and emergency C-sections were included. Furthermore, some of the bitches selected for the elective procedure had serum progesterone levels lower than 2 ng/mL. In the light of the association between serum progesterone concentration and cervical opening [25], the present research included only bitches with a serum progesterone concentration higher than 2 ng/mL and queens that had not shown any sign of impending parturition, to avoid possible ascending contamination via the vagina. Compared to previous investigations [8,19], this research had stricter inclusion criteria and negative laboratory and environmental controls were added: all pregnancies were healthy, C-sections were elective, and more samples were included (i.e., swabs from the surgical tray) to assess environmental contamination.

Both culture and sequencing resulted in detection of bacteria but, despite the strict procedures that were adopted, a critical analysis of both culture and sequencing data suggests that contamination is still the most likely source. All the bacteria species isolated via culture in the present research are ubiquitous and commonly found in the environment. In fact, both coagulase negative staphylococci and *Bacillus* spp. are known to be common environmental contaminants in microbiology laboratories [31]. Bacteria belonging to the genus *Acinetobacter* are ubiquitous [32]; specifically, *Acinetobacter baumannii* is renowned for its presence in hospitals, where it can be responsible for nosocomial infections [33] as well as *Pseudomonas aeruginosa* [34]. Finally, *Pseudomonas fluorescens* optimally grows in substrates such as disinfectants [35]. The presence of *S. epidermidis* and *S. hominis* was detected either in the placenta or in the meconium of newborn puppies by Zakošek Pipan (2020), in a study that included natural births and emergency C-sections [19]. Coagulase-negative staphylococci were isolated from the amniotic fluid of horses [36], along with *Acinetobacter* spp., which was also reported in bovine feto-maternal elements [7]. However, contamination cannot be ruled out due to the inclusion of emergency C-sections or natural birth [9,19] and due to the high abundance of some of these genera in laboratory reagents (e.g., DNA extraction kit) [7].

The genetic material of the bacteria that were isolated in culture was not always identified using molecular techniques, leading us to hypothesize that contamination could have occurred during culture seeding or incubation. Bacteria that were found through both culture and sequencing (54.5%) were not abundant (absolute reads count ranging from 4 to 189 reads and relative abundance ranging from 0.8 to 40%) and, as mentioned, were ubiquitous microorganisms. The bacterial abundance detected using molecular analyses in all animal samples (from uterus, amniotic fluid, and meconium) was lower than the abundance found in environmental swabs from the surgical tray, which served as a negative control for the sampling procedures, being sterile by definition [37], and always yielding negative cultures in the present study. This means that the pregnant uterus of small animals has a very low bacterial load, approaching the sterile womb hypothesis. Interestingly, sequencing results showed a species-specific pattern. In fact, bacterial populations resulting from feline and canine samples grouped into two clusters, matching the species of origin. This cannot be linked to cross-contamination during laboratory processing because canine and feline samples were sequenced in the same run. We may hypothesize that this in-between species difference is related to the characteristic mucosal and skin microbiota of dogs and cats [38,39] that enters the surgical room via its host. Similarly, in humans, C-section-born neonates harbor bacterial communities that resemble those of the skin of the mother [40]. However, we acknowledge that the limited sample size of the present preliminary study does not allow us to draw definitive conclusions, and that further research is needed.

The microbial profile of canine and feline amniotic fluid, uterus, and meconium resulting from 16S rRNA sequencing was dominated by Bacteroidetes, Firmicutes, and Proteobacteria in different proportions. However, no relevant difference was found among tissues at lower taxonomic levels and the same phyla were also prevalent in the environmental controls. Overall, these results were consistent with those of previous studies in other species [7,16,41]. Interestingly, some sequences belonging to anaerobic bacteria (i.e., belonging to the Phylum Fusobacteria) were identified using molecular techniques in very low abundance. These were never isolated in culture, although the samples were collected and transported using specific swabs and media intended to preserve the viability of aerobes, anaerobes, and fastidious bacteria (Copan eSwab with Amies medium, Copan, Italy), and anaerobic culture was performed. However, the presence of these bacterial sequences in multiple samples can hardly be associated with contamination from the animal skin of from the environment, as these bacteria are strict anaerobes. If the feto-maternal samples are the source of this genetic material, it probably resulted from dead microorganisms, as no isolation in culture occurred. This observation may be consistent with the protective role of the placental barrier and amniotic fluid against possible pathogens [1]. If this hypothesis were confirmed, it could explain the presence of genetic material belonging to other non-viable bacteria. Next-Generation Sequencing (NGS) techniques have the advantage of detecting all bacteria, including the unculturable ones [41], or those that require very specific media and long incubation times to grow [42]. Still, molecular techniques act as a double-edged sword, and the presence and significance of bacteria can be overestimated. NGS techniques detect bacterial DNA without discriminating between living and dead microorganisms and between sequences deriving from cross-contamination (during sampling and/or DNA extraction) and biologically relevant ones. Furthermore, low biomass samples often contain the same amount of DNA and similar alpha diversity (within-sample diversity) when compared to controls, not allowing us to discriminate whether the genetic material originates from the animal samples or from the environment. Contamination is a major challenge when investigating feto-maternal microbiome and low-biomass samples in general [1,17,18], and the sampling techniques used in most studies in humans and veterinary species did not guarantee sterility [9,19,43,44]. In the present study, we tend to exclude the environment of the surgical room as a source of contamination because samples were collected in two different facilities, but this did not influence either the results obtained via culture nor via sequencing. The coat, skin, and mucosae of the animals undergoing C-sections may represent the source of contamination. The hypothesis that contamination originated from the animal itself and not from the surgical room is also supported by the negative culture results for all environmental controls. This, along with bacteria selection related to culture media [45], could explain the presence of bacteria. Future research should include more controls, including swabs of the skin of the mother, the abdominal serosa, and the gloves of the surgeon, to better define the possible source of contamination.

## 5. Conclusions

This is the first study investigating feto-maternal microbiota in domestic carnivores using a combination of techniques and implementing measures for strict asepsis, from animal selection, sampling procedures, and inclusion of controls. Furthermore, to the best of the authors’ knowledge, this study represents pioneering research on feline feto-maternal microbiota.

In the framework of the debate concerning the in utero colonization of newborns, the results of the present study add more knowledge about the microbial presence within the fetal environment of domestic carnivores. Regardless of the preliminary nature of this research and the small number of animals included, we suggest that a very low load of bacterial genetic material of unknown viability can be found during healthy pregnancies at term.

## Figures and Tables

**Table 1:** Age, weight, litter size, and list of breeds of the bitches and queens included in the study.

	Age Years Mean ± SD	Weight kg Median (Range)	Litter Size Mean ± SD	Breed
Bitches	3.8 ± 2.2	38 (3.2–63)	5 ± 1.4	Boston Terrier, Chihuahua, Dogue de Bordeaux, English Staffordshire Bull Terrier, French Bulldog
Queens	4.5 ± 2	3.9 (3.3–4.3)	2.5 ± 0.5	Russian blue (*n* = 2), Scottish fold

**Table 2:** Bacteria isolation from five canine feto-maternal units (C-FMU): uterus (placental site), amniotic fluid, and meconium of the first extracted fetus: results of isolation are reported, together with the isolated bacterial species and the growth * based on the number of colony-forming units (CFUs) in the first isolation plates.

	Uterus ^a^			Amniotic Fluid ^b^			Meconium ^c^		
FMU	Result	Bacteria	Growth *	Result	Bacteria	Growth *	Result	Bacteria	Growth *
C-FMU-1	neg			neg			neg		
C-FMU-2	+	*Bacillus* spp. ^X^	High	+	*Bacillus* spp.	High	+	Coagulase-negative Staphylococcus (*S. hominis*) ^X^ *Acinetobacter baumannii* ^X^	High High
C-FMU-3	neg			neg			+	Coagulase-negative Staphylococci (*S. epidermidis*)	Low
C-FMU-4	neg			neg			neg		
C-FMU-5	+	*Pseudomonas* spp. (*p. fluorescens*)	Low	neg			+	*Acinetobacter lwoffii* ^X^	High

^a^ Site of attachment of the placenta to the endometrium of the first extracted fetus; ^b^ amniotic fluid of the first extracted fetus; ^c^ meconium sampled from the rectal ampulla of the first extracted fetus; ^X^ the presence at genus level was confirmed by sequencing. * Low (1–10 CFU/10 µL), Moderate (11–30 CFU/10 µL), or High (≥31 CFU/10 µL).

**Table 3:** Bacteria isolation from three feline feto-maternal units (F-FMU): uterus (placental site), amniotic fluid, and meconium of the first extracted fetus: results of isolation are reported, together with the isolated bacterial species and the growth * based on the number of colony-forming units (CFUs) in the first isolation plates.

	Uterus ^a^			Amniotic Fluid ^b^			Meconium ^c^		
FMU	Results	Bacteria	Growth *	Result	Bacteria	Growth *	Result	Bacteria	Growth *
F-FMU-1	neg			neg			neg		
F-FMU-2	+	*Coagulase-negative Staphylococcus (S. epidermidis)*	** Very low *	+	*Pseudomonas aeruginosa* ^X^	** Very low	neg		
F-FMU-3	+	*Pseudomonas aeruginosa* ^X^	High	neg			+	*Psychrobacter sanguinis*	** Very low

^a^ Site of attachment of the placenta to the endometrium of the first extracted fetus; ^b^ amniotic fluid of the first extracted fetus; ^c^ meconium sampled from the rectal ampulla of the first extracted fetus; ^X^ the presence at genus level was confirmed by sequencing. * Low: (1–10 CFU/10 µL), Moderate (11–30 CFU/10 µL), or High (≥31 CFU/10 µL); ** Very low: bacterial cultures obtained from seeding of broth cultures (growth only from enrichment broth HIB, not on first isolation media).

**Table 4:** Association in bacterial strain at order, family, genus, and species levels between canine and feline feto-maternal units, feto-maternal tissues and controls, surgical facility, and laboratory run procedure. *p* values of (a) Chi-square test and (b) Fisher’s exact test indicate the associations between the cluster membership and the grouping method.

Grouping Method	K ^1^	Order *p*-Value	Family *p*-Value	Genus *p*-Value	Species *p*-Value
Animal species	(K = 2)	0.017 ^a^ ** 0.008 ^b^ ***	0.033 ^a^ ** 0.020 ^b^ **	0.062 ^a^ 0.057 ^b^	0.033 ^a^ ** 0.020 ^b^ **
Feto-maternal unit	Cats (K = 3)	0.45 ^a^ 0.68 ^b^	0.73 ^a^ 1 ^b^	0.38 ^a^ 0.63 ^b^	0.34 ^a^ 0.59 ^b^
	Dogs (K = 5)	0.59 ^a^ 0.68 ^b^	0.52 ^a^ 0.60 ^b^	0.58 ^a^ 0.71 ^b^	0.61 ^a^ 0.82 ^b^
Feto-maternal tissues vs. controls	Cats (K = 2)	0.82 ^a^ 0.54 ^b^	1 ^a^ 1 ^b^	1 ^a^ 1 ^b^	1 ^a^ 1 ^b^
	Dogs (K = 2)	0.51 ^a^ 0.53 ^b^	0.51 ^a^ 0.53 ^b^	0.51 ^a^ 0.53 ^b^	0.43 ^a^ 0.31 ^b^
Tissues (uterus, amniotic fluid, meconium, controls)	Cats (K = 4)	0.46 ^a^ 0.77 ^b^	0.47 ^a^ 0.78 ^b^	0.34 ^a^ 0.58 ^b^	0.22 ^a^ 0.18 ^b^
	Dogs (K = 4)	0.11 ^a^ 0.25 ^b^	0.035 ^a^ ** 0.09 ^b^	0.07 ^a^ 0.08 ^b^	0.35 ^a^ 0.34 ^b^
Surgical facility	(K = 2)	0.90 ^a^ 0.70 ^b^	0.74 ^a^ 0.67 ^b^	1 ^a^ 1 ^b^	0.73 ^a^ 0.67 ^b^

^1^ K indicates the value of k clusters recognized during k-means analysis; ^a^ Chi-squared test p; ^b^ Fisher’s Exact test p; ** and *** show *p* < 0.05 and *p* < 0.01, respectively.

## Data Availability

BioSample metadata are available in the NCBI Biosample database (http://www.ncbi.nml.nih.gov/biosample/ (accessed on 1 May 2023)) under accession number SUB11494484 (BioProject ID PRJNA841953).
